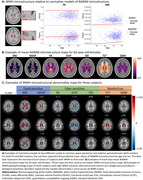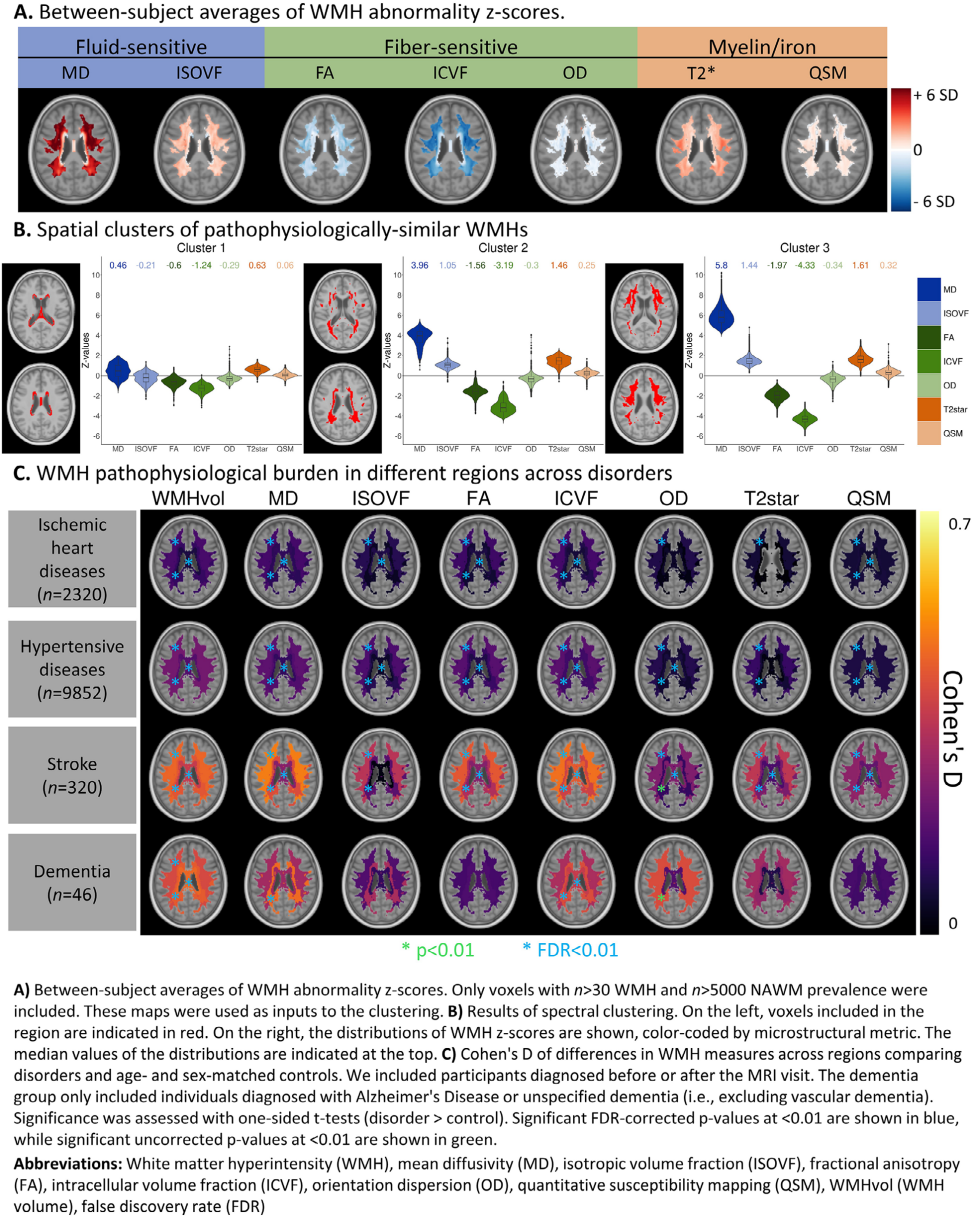# Spatial Characterization of White Matter Hyperintensity Pathophysiology Across Disorders

**DOI:** 10.1002/alz.085913

**Published:** 2025-01-09

**Authors:** Olivier Parent, Gabriel A. Devenyi, Aurelie Bussy, Grace Pigeau, Manuela Costantino, Jérémie Fouquet, Daniela Quesada Rodriguez, Mahsa Dadar, Mallar M. Chakravarty

**Affiliations:** ^1^ McGill University, Montreal, QC Canada; ^2^ Douglas Mental Health University Institute, Montreal, QC Canada

## Abstract

**Background:**

White matter hyperintensities (WMHs) are age‐related radiological abnormalities indicative of small vessel disease. It is unclear if WMHs in different regions represent similar pathophysiology and etiology. Here, we developed a framework to estimate WMH pathophysiology in vivo, which allowed us to precisely characterize spatial patterns of WMH tissue alterations associated with four disorders.

**Method:**

We used data from 32,014 UK Biobank (UKB) participants. WMHs and normal‐appearing white matter (NAWM) were automatically segmented. Diffusion‐ and susceptibility‐weighted images were used to derive fluid‐, fiber‐, myelin‐ and iron‐sensitive microstructural markers. We calculated voxel‐wise normative models of NAWM microstructure using Bayesian linear regression with age (4th order B‐spline) and sex predictors in a custom UKB template space derived using multi‐spectral registration of T1w and fractional anisotropy (FA) images (Figure 1A‐B). Within‐subject WMH pathophysiology was estimated as the difference between WMH microstructure and predicted NAWM microstructure (Figure 1C).

**Result:**

First, from between‐subject averages of WMH microstructural abnormality (Figure 2A), we used spectral clustering to derive spatial patterns of WMHs that share similar pathophysiological properties (Figure 2B). The first cluster (periventricular) has low abnormality. The second (posterior) and third (anterior) cluster both show fluid accumulation, fiber alterations, and myelin and iron loss, while the anterior cluster shows higher abnormality. Second, we assessed the differences between four disorders and age‐ and sex‐matched controls in terms of all WMH features with Cohen’s D. Ischemic heart diseases (*n*=2320) and hypertensive diseases (*n=*9852) demonstrated small but significant effects across measures, which were slightly higher in anterior regions. Stroke (*n*=320) demonstrated larger effects, which were more prominent in anterior regions. On the other hand, dementia (*n*=46) demonstrated smaller effects compared to stroke, reaching significance mostly in posterior regions despite the smaller sample size. Interestingly, the fractional anisotropy and orientation dispersion markers showed drastically inverted patterns of effects between stroke and dementia.

**Conclusion:**

Our results separating anterior and posterior WMHs are consistent with accumulating evidence showing that posterior WMHs may be linked to Alzheimer’s pathology, whereas anterior WMHs may be associated with vascular pathologies. MRI pathophysiological markers may further help in distinguishing these etiologies.